# Ultrasensitive SERS immunoassay based on diatom biosilica for detection of interleukins in blood plasma

**DOI:** 10.1007/s00216-017-0566-5

**Published:** 2017-08-29

**Authors:** Agnieszka Kamińska, Myroslav Sprynskyy, Katarzyna Winkler, Tomasz Szymborski

**Affiliations:** 1 Institute of Physical Chemistry, Polish Academy of Sciences, Kasprzaka 44/52, 01-224 Warsaw, Poland; 2 Department of Environmental Chemistry and Bioanalytics, Faculty of Chemistry, Nicolaus Copernicus University, 7 Gagarina Str, 87-100 Toruń, Poland

**Keywords:** Surface-enhanced Raman spectroscopy (SERS), Interleukin 8, Diatom biosilica, Immunoassay

## Abstract

An ultrasensitive surface-enhanced Raman scattering (SERS) immunoassay based on diatom biosilica with integrated gold nanoparticles (AuNPs) for the detection of interleukin 8 (IL-8) in blood plasma has been developed. The SERS sensing originates from unique features of the diatom frustules, which are capable of enhancing the localized surface-plasmon resonance of metal nanostructures. The SERS immune tags ware fabricated by functionalizing 70-nm Au nanoparticles with DTNB (i.e., 5,5′-dithiobis(2-nitrobenzoic acid)), which acted as a Raman reporter molecule, as well as the specific antibodies. These DTNB-labeled immune-AuNPs can form a sandwich structure with IL-8 antigens (infection marker) and the antibodies immobilized on the biosilica material. Our method showed an improved IL-8 detection limit in comparison to standard ELISA methods. The current detection limit for IL-8 using a conventional ELISA test is about 15.6 pg mL^−1^. The lower detection limit for IL-8 in blood plasma was estimated to be 6.2 pg mL^−1^. To the best of our knowledge, this is the first report on the recognition of IL-8 in human samples using a SERS-based method. This method clearly possesses high sensitivity to clinically relevant interleukin concentrations in body fluids. The average relative standard deviation of this method is less than 8%, which is sufficient for analytical analysis and comparable to those of classical ELISA methods. This SERS immunoassay also exhibits high biological specificity for the detection of IL-8 antigens. The established SERS immunoassay offers a valuable platform for the ultrasensitive and highly specific detection of immune biomarkers in a clinical setting for medical diagnostics.

**Electronic supplementary material:**

The online version of this article (10.1007/s00216-017-0566-5) contains supplementary material, which is available to authorized users.

**Graphical Abstract:**
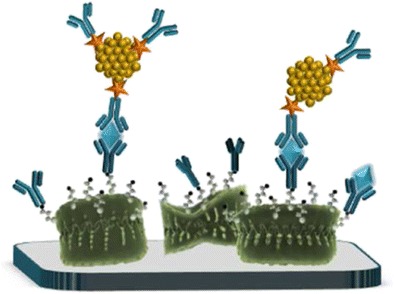
The SERS-based immunoassay based on naturally generated photonic biosilica for the detection of interleukin 8 (IL-8) in human plasma samples

## Introduction

Interleukins are secreted proteins that are members of the cytokine family of immune system molecules that regulate immune cell activity. Interleukins are produced by immune system cells such as lymphocytes, macrophages, and monocytes [1]. They modulate inflammation and immunity by regulating the growth, mobility, and differentiation of lymphoid and other cells [2]. The analysis and quantitation of interleukins in body fluids is important as it allows us to broaden our understanding of their immunological functions. Cytokine levels in body fluids can provide information useful for disease diagnosis and staging, prognostication, and thus the selection of an appropriate disease therapy [3]. Several analytical procedures for quantifying interleukins in body fluids and tissue culture supernatants have been developed. Enzyme-linked immunosorbent assays (ELISA) are the most popular methods of quantitating secreted cytokines due to their high specificities and sensitivities [4, 5]. Intracellular staining, the ribonuclease protection assay (RPA) [6], the polymerase chain reaction (PCR) [7], and cytometric assays [8] have also been used in recent decades. However, each of these techniques has at least one significant limitation. For instance, problems with cytokine assays, including a lack of accuracy, have been reported; a number of factors have been shown to affect the validity and quality of measurements obtained with such assays [9–12].

Therefore, there is a need to develop a more sensitive, selective, stable, and durable method for analyzing these biomarkers. Recent advances in nanotechnology and instrumentation development have permitted the development of a highly sensitive and chemically specific technique for biomolecular system recognition that uses surface-enhanced Raman spectroscopy (SERS) [13]. The phenomenon of SERS can be explained as a combination of an electromagnetic mechanism (EM) and a chemical mechanism related to charge transfer between a substrate and an adsorbed molecule [14]. Theoretically, the electromagnetic enhancement can reach factors of 10^3^ to 10^11^, whilst chemical enhancement factors of up to 10^3^ have been calculated [15, 16]. This huge enhancement in Raman scattering—even single molecules can be observed—ensures that Raman spectroscopy is very effective for ultrasensitive bioanalysis [17]. Another interesting characteristic of SERS is the linear dependence of the SERS intensity on the power of the incident light, despite the nonlinear signal enhancement that is achieved with this technique. Thus, SERS could potentially be applied for the quantitative measurement of analytes with ultrahigh sensitivity.

SERS biosensing has been used to detect various biological samples and diseases, including various cancers [18], Alzheimer’s disease [19], and Parkinson’s disease [20]. The most notable recent advances in SERS include the application of this technique to immunosensing. SERS-based immunoassays have attracted significant research interest due to their (i) high detection sensitivities and selectivities [21], (ii) reduced susceptibility to photobleaching [21], and (iii) narrow spectral bandwidth, allowing multiplex analysis [22].

Wang et al. [23, 24] demonstrated the detection of the interleukins IL-6 and IL-8 from buffer solution. Typically, SERS immunoassays are realized on flat glass, on a noble metal (Au, Ag, or alloys of them) surface with nanoscale roughness [25], or on photonic crystals [26]. Unfortunately, silver—which gives the best signal enhancement factors—undergoes oxidation, so when a silver surface is used, SERS must be carried out before the surface oxidizes. Photonic crystals, despite their excellent properties, are expensive to develop as techniques requiring high-tech equipment (i.e., PVD sputtering, photolithography, focused ion beam, and others) are needed.

In the work reported in the present paper, we investigated a more accessible approach based on natural photonic crystals (materials) such as diatom biosilica. Diatoms are photosynthesizing algae that possess a siliceous skeleton called a frustule [27] comprising complex hierarchical micro- to nanosized structures under natural conditions. We employed diatom silica frustules produced by *Pseudostaurosira trainorii* as an inexpensive and easy to prepare and modify functional material to use in a novel SERS immunoassay.

Obtaining three-dimensional (3D) structures of inorganic materials is one of the main challenges involved in the development of nanotechnology. The shape and the pattern of a frustule are unique to the particular diatomic species that produced it [28]. Thus, highly individual 3D silica structures can be obtained from single-celled diatoms without the need to use complex and expensive nanofabrication methods. Diatom frustules exhibit unpredictable optical properties due to their quasi-ordered pore patterns, such as diffraction-driven self-focusing [29] and gas-sensitive photoluminescence emission [30]. Researchers have investigated whether the unusual optical properties of frustules could be used in practical applications. Gale et al. [30] demonstrated the use of an antibody-functionalized diatom biosilica frustule as a microscale biosensor platform for the selective and label-free photoluminescence-based detection of biomolecules. Kong et al. [31] fabricated a photonic biosilica SERS substrate by integrating Ag NPs into microchannels of diatom frustules to identify explosive molecules in nanoliter solutions.

To the best of our knowledge, the present paper represents the first report of the application of a SERS immunoassay based on diatom biosilica to the detection of interleukin 8 (IL-8) in human blood plasma. IL-8 is an inflammatory cytokine that also plays an important role in breast cancer. There have been a few studies of the biological activity of this cytokine; for instance, Yokoe et al. [32] measured serum IL-8 in 12 heavily pretreated patients with recurrent breast cancer, and reported that IL-8 levels were higher in patients with refractory progressive disease but were almost unchanged in patients showing a partial response or no change after systemic therapy. In this paper we demonstrate the use of diatom biosilica as a SERS immune substrate.

## Methods

### Reagents

Recombinant human interleukin 8 (CXCL8) and monoclonal anti-interleukin-8 antibody produced in mouse (clone 6217) were purchased from Sigma (St. Louis, MO, USA) and used as received. 5,5′-Dithiobis(2-nitrobenzoic acid) (DTNB), L-ascorbic acid, gold(III) chloride trihydrate, trisodium citrate dihydrate, hexadecyltrimethylammonium bromide (CTAB), aminopropyltriethoxylsilane (APTES), bovine blood plasma albumin (BSA), and phosphate-buffered saline (PBS) packs (10 mM, pH 7.2) were also obtained from Sigma.

### Blood sample preparation

In our experiments, we used human blood samples from 10 healthy volunteers. These samples were made available courtesy of the Regional Blood Center (Warsaw, Poland). The samples underwent morphological analyses prior to use and revealed no abnormalities. All experiments were performed in compliance with the relevant laws and institutional guidelines. The study protocol was approved by the Ethics and Bioethics Committee of Cardinal Stefan Wyszynski University in Warsaw.

### Fabrication of diatom biosilica substrates

**Fig. 1:**
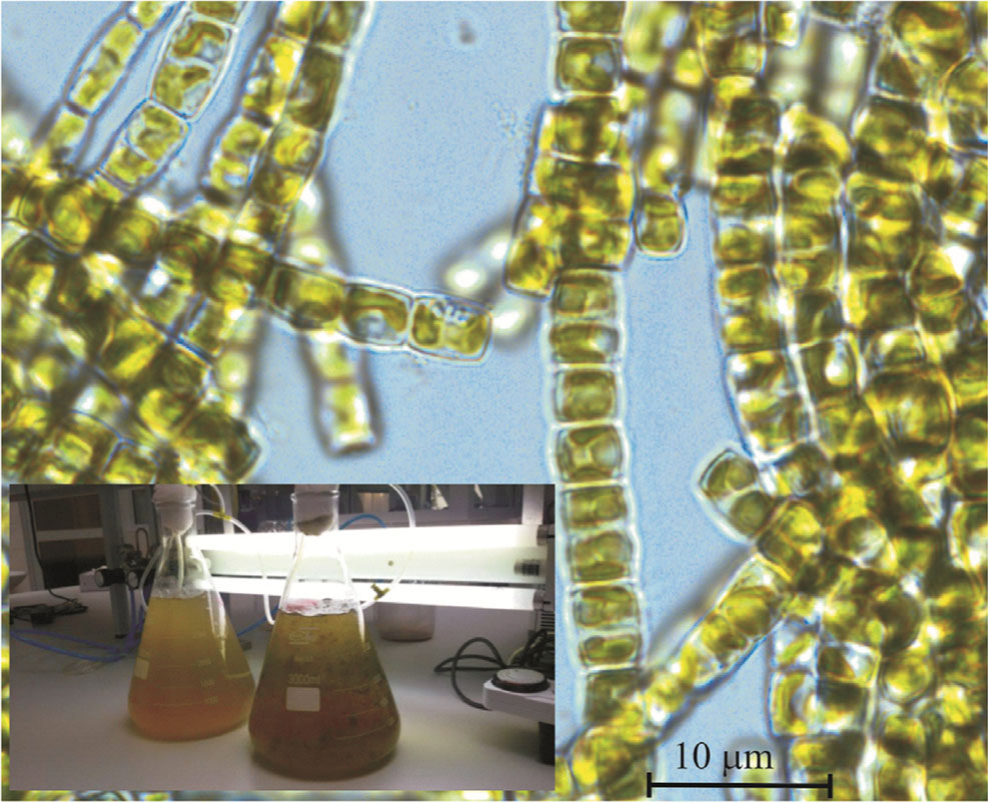
Images of the algae cultivation setup and colonial chains of living diatoms

A culture of the diatom species (*Pseudostaurosira trainorii*) was cultivated using Erlenmeyer flasks with f/2 nutrient solution containing silica at a concentration of 7 mg mL^−1^ under aeration and a 12 h light/12 h darkness regime. The light was provided by two fluorescent lamps with an intensity of 1500 lx. The setup used for algae cultivation and a light microscopy image of living diatoms in the form of long colonial chains are shown in Fig. 1. After they had grown, the diatoms were washed out and treated with hydrogen peroxide in order to isolate silica frustules from the organic cellular matter according to the procedure described by Yang et al. [33]. The production capacity of the diatom species was about 320 mg (dry weight) per liter.

### Fabrication of the immune platform

Diatom frustules are mainly composed of amorphous hydrated SiO_2_ and are thus amenable to simple chemical functionalization for multiple applications (e.g., in photonics and analytical chemistry). To make the diatom frustules (biosilica) useful for immune biosensing, glass slides were modified with the frustules, which was achieved as follows. The biosilica was dispersed in ethanol at a concentration of 0.5 mg mL^−1^. The cleaned glass slides were covered with 100, 300, 800, and 1000 μl of this diatom biosilica solution, respectively. All the samples were placed in an oven for 1 h at 450 °C. In the next step, the surface silanol groups of the biosilica were functionalized with amine groups using aminopropyltriethoxylsilane (APTES) according to the procedure described by Yang et al. [33]. The glass slides with diatom frustules were incubated in 3 mL of a 1% solution of APTES in ethanol for 1 h at 50 °C. The substrates were then removed from solution and rinsed with ethanol and DI water after 5 min. The amino-modified surfaces were functionalizedd with anti-interleukin-8 (anti-IL-8) antibodies by immersing the frustules in a mixture of 25 μL of 150 μg mL^−1^ anti-IL-8 in phosphate-buffered saline (PBS) and 5μL of activation solution (0.2 M EDC/0.05 M NHS; mixture in deionized water). Covalent bonds were formed between activated carboxyl groups on the anti-IL-8 antibodies and free amino groups on the diatoms. After 1 h, the substrates with immobilized antibodies were rinsed with DI water and dried with nitrogen gas. Remaining active surface groups were blocked by immersing the substrates in 15 μL of 2% BSA in PBS buffer solution (pH 7.2). Finally, the samples were rinsed twice with 5 mL of 10 mM PBS buffer solution and stored at 4 °C in PBS for future use.

### Synthesis of Au nanoparticles

Gold nanoparticles 70 nm in diameter (see Fig. S1 in the “Electronic supplementary material,” ESM) and capped with CTAB (AuNPs@CTAB) were obtained via a two-step seeding protocol [34]. In the first step, AuNPs 15 nm in diameter were synthesized using the Turkevich approach [35]. In this approach, seeds were obtained by first adding HAuCl_4_.3H_2_O (49 mg, 0.125 mmol) to 250 mL of boiling water and then adding trisodium citrate dihydrate (125 mg, 0.425 mmol). In a second step, CTAB (2.74 g, 7.5 mmol) was dissolved in 500 mL of water heated to 35 °C. A solution of HAuCl_4_.3H_2_O in water (2.5 mL, 0.1 M) was then added and stirring was continued until the mixture became clear. Next, a solution of ascorbic acid (2 mL, 2.5 mM) was injected and, after complete discoloration of the reaction mixture, 6.5 mL of the seed solution were added quickly. The mixture was stirred gently for 1 h and then centrifuged and carefully decanted. The precipitate was dispersed in 8 mL of CTAB water solution (0.1 M) and left overnight to allow the shape-selective separation of nonspherical from spherical particles formed during the growing process. The solution of spherical AuNPs@CTAB was then carefully collected from above the sediment of unwanted nonspherical particles. The concentration of gold atoms in the final solution, as determined from the absorption spectrum, was 15.42 mM.

### Synthesis of anti-IL18/AuNPs-DTNB

The solution of AuNPs@CTAB (1 mL) was centrifuged (6000 rpm, 10 min) in order to remove excess CTAB. The supernatant was carefully decanted and the precipitate was dissolved in water (1 mL). A solution of DTNB (18 mg, 0.045 mmol, in 5 mL of ethanol) was added during stirring and the obtained mixture was left overnight. The AuNPs@DTNB were collected by centrifugation (2000 rpm, 10 min) and purified by sixfold dissolution in acetonitrile, precipitation with methanol, and centrifugation (6000 rpm, 10 min) and then by tenfold dissolution in water and centrifugation (7500 rpm, 10 min). The purified AuNPs were dried and suspended in 2.5 mL of water. Next, the 10 μL of solution containing the DTNB-modified Au nanoparticles were mixed with the solution of anti-IL8 in PBS buffer (5 μL, 60 μg mL^−1^). For the synthesis of conjugate, the coupling reagents (0.2 M EDC/0.05 M NHS; in the volume ratio of 5:1, in deionized water) were added and then the resulting mixture was incubated at 4 °C for 4 hours. The Raman-reporter-labeled immune Au nanoparticles (anti-IL8/AuNPs-DTNB) were separated from the solution by centrifugation at 10,000 rpm for 5 min. The suspensions of anti-IL8/AuNPs-DTNB were then passivated with 2.5 μL of 2% BSA in PBS buffer solution. After 2 h, the mixture was centrifuged again for 10 min at 20,000 rpm and then re-suspended in 1 mL of the PBS solution. The prepared anti-IL8/AuNPs-DTNB were stored at 4 °C for future use. The maximum number of antibodies bound to a nanoparticle was estimated to be ca. 540 [36]. The average diameter of a gold nanoparticle was about 70 nm according to SEM images and a histogram of Au nanoparticle diameters (ESM Figs. S1 and S2).

### Immunoassay protocol

**Fig. 2:**
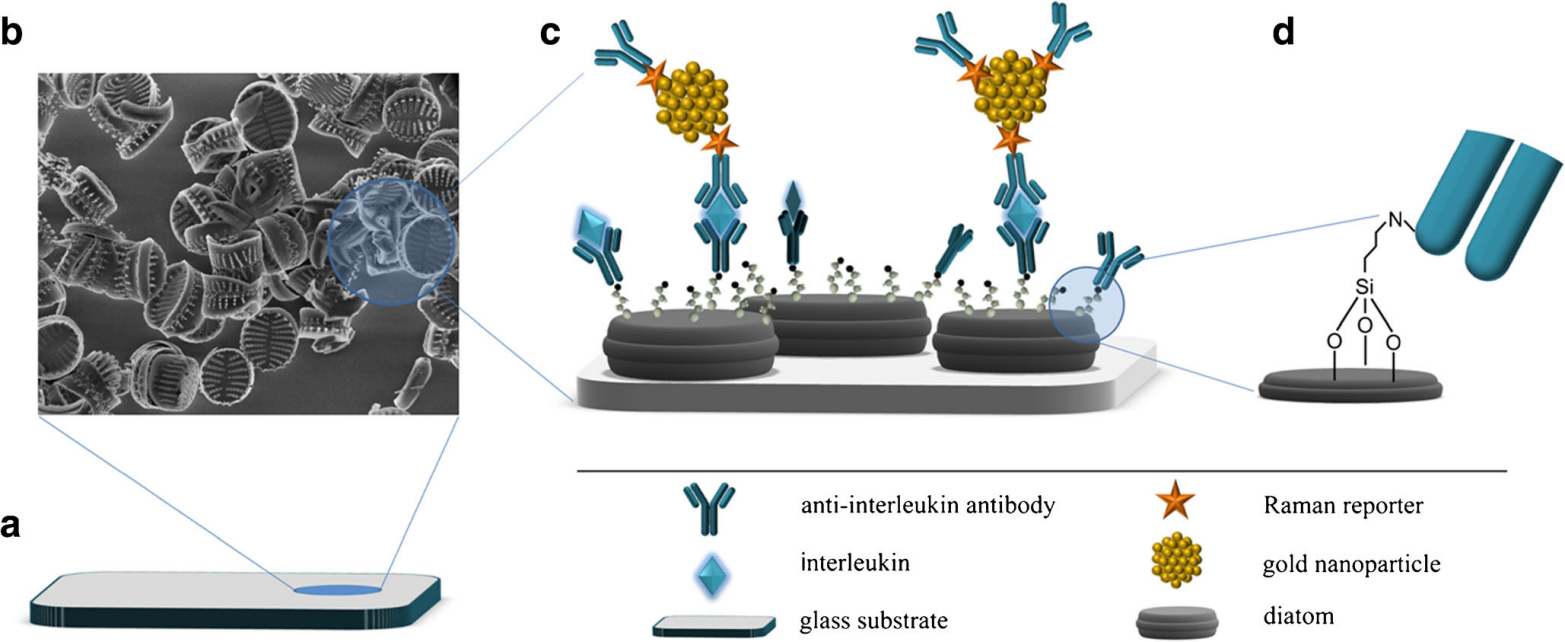
**a–d** Schematic illustration of the SERS-based immunoassay: **a** glass slide was modified with diatom frustules (**b** shows an SEM image of a portion of the modified slide); **c** antibody capture and immobilization using the SERS immunosensor for interleukin 8 detection (**d** shows a close-up illustrating the bonding to the antibody)

Immune reactions between antigens and antibodies were performed in a sandwich-type SERS immunoassay, as schematically illustrated in Fig. 2.

The first layer of this sandwich structure is composed of immobilized antibodies against IL-8 captured on the amino-modified biosilica substrate. The second layer contains the complementary interleukins (proteins) captured by these selective antibodies. The third layer consists of Raman reporter DTNB-labeled immune Au nanoparticles. The presence of IL-8 in the analyzed samples is identified through the appearance of the SERS spectrum of the DTNB bonded with the specific antibody against the studied interleukin.

#### Washing conditions

To limit nonspecific binding interactions, the immunoassay was washed three times with PBS buffer solution (pH 7.2).

## Instrumentation

### Raman and SERS measurements

SERS measurements were performed using a Renishaw (Wotton-under-Edge, UK) inVia Raman system equipped with a 632-nm He-Ne laser excitation source. The light from the laser was passed through a line filter and focused on a sample mounted on an *x*-*y*-*z* translation stage with a 50× microscope objective (N.A. = 0.75). The Raman-scattered light was collected by the same objective through a holographic notch filter to block out Rayleigh scattering. A grating with 1800 grooves per mm was used to provide a spectral resolution of 5 cm^−1^. The Raman scattering signal was recorded by a 1024 × 256 pixel RenCam CCD detector. The beam diameter was approximately 5 μm. Typically, the SERS spectra were recorded over an integration time of 30 s with a laser output power of 2.5 mW by mapping an area of size 50 μm × 50 μm.

### Electron microscopy characterization

The morphological and structural features of the cleaned diatom frustules were examined by high-resolution scanning electron microscopy (SEM). SEM measurements of diatoms on the glass slide were done under high vacuum using the FEI (Hillsboro, OR, USA) Nova NanoSEM 450 with an accelerating voltage of between 2 and 30 kV. The glass slide was attached to the SEM table with conductive silver paste.

The elemental composition of the diatom frustules was determined using transmission electron microscopy (FEI Tecnai F20 X-Twintool) coupled with energy-dispersive X-ray detection (EDX), with the sample being placed on a carbon-coated copper grid (lacey carbon support film, 400 mesh). In the study, samples of diatom frustules immobilized on glass slides and diatom frustules dispersed in ethanol were used.

## Results and discussion

### Characterization of the substrate and Raman-reporter-labeled immune Au nanoparticles

According to research results obtained by Sprynskyy et al. [37], the examined biosilica can be characterized as a macroporous material that contains a network of meso- and micropores with a BET surface area of 16.9 m^2^/g. The pores showed size ranges of 1–1.5 nm, 10–20 nm, and 25–70 nm. The biosilica was identified as opal-A by X-ray diffraction and was termed “naturally organic functionalized 3D silica” due to the presence of residual organic functional groups related to biosilica-associated proteins as well as the functional groups characteristic of the amorphous silica framework. Also, the biosilica exhibited photoluminescence in the mid-ultraviolet region as well as in the blue-green region of the visible spectrum [37].

**Fig. 3:**
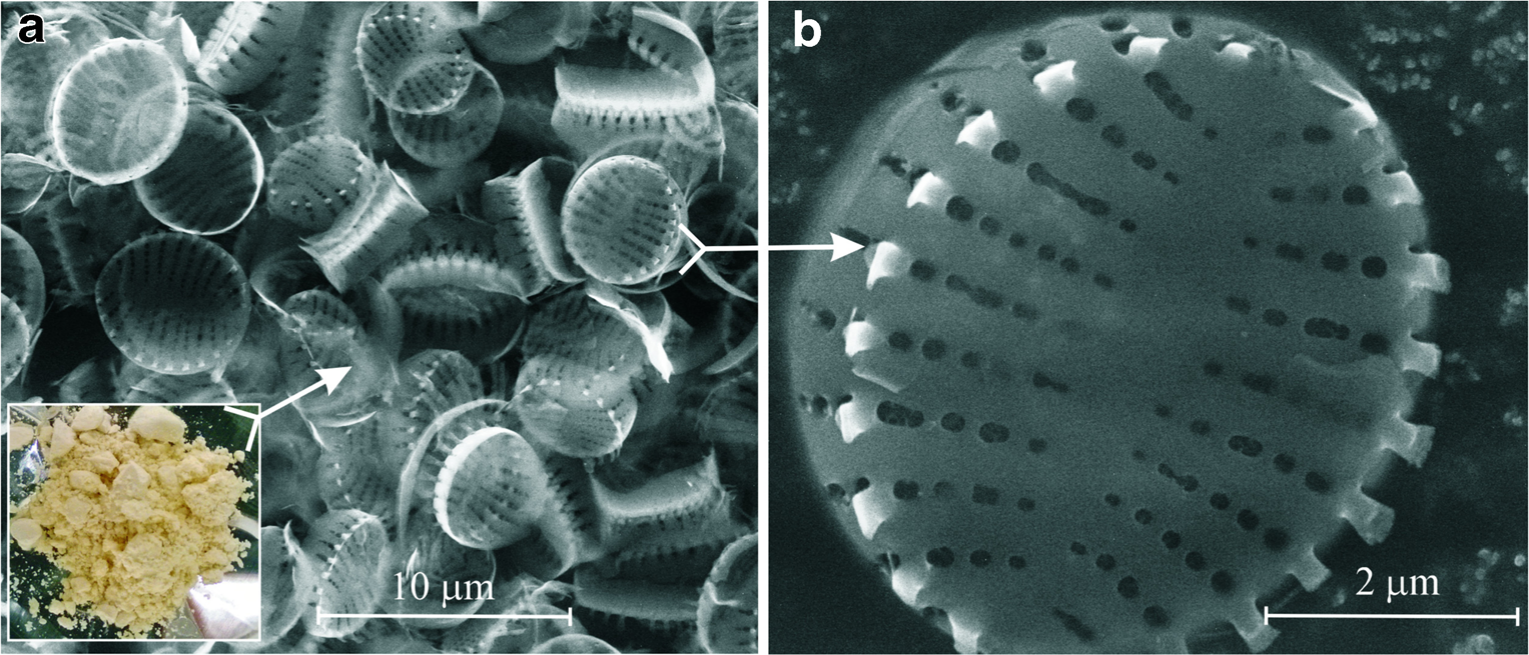
**a–b** SEM images of a diatom frustule assembly (**a**) and view of a single diatom valve from above (**b**)

Scanning electron microscopy images of the diatom frustules used in our studies, which were isolated from cultivated diatoms of *Pseudostaurosira trainorii*, are presented in Fig. 3.

Figure 3a shows an assembly of uniform and well-preserved diatom frustules. The external and internal surfaces as well as sidewall regions of the diatom valves can also be observed. It is clear that the valves possess round to slightly elliptical forms with an average diameter of 4–5 μm. The inset in Fig. 3a shows a macroscopic image of the dried diatom frustules. Figure 3b reveals the intricate design of the external surface of a single diatom valve. The valve surface is split in two halves axially by a solid strip (raphe), with each half perforated by parallel linear rows of oval pores (areolae). The rows of pores are oriented perpendicular to the central axis and each contain 4–5 pores of size 100–200 nm. The pore size decreases towards the central axis. Neighboring rows are separated by about 450 nm and neighboring pores in a row are separated by about 100 nm. TEM-EDX analysis (ESM Fig. S3) showed that the cleaned diatom frustules were 98% silica. The dominant peaks in the TEM-EDX spectrum of the frustules were from silicon and oxygen. The low O/Si atomic ratio (1.92) observed is characteristic of an amorphous hydrated silica (such as opal). Peaks attributed to Cu derive from the copper TEM grid employed. The detection of carbon indicated the presence of strongly bound residual organics in the cleaned diatom frustules. Furthermore, the initial iron concentration in the nutrient solution was very low: 0.65 mg L^−1^, and the concentrations of other trace elements (Zn, Cu, Co) in the nutrient solution were an order of magnitude lower still.

**Fig. 4:**
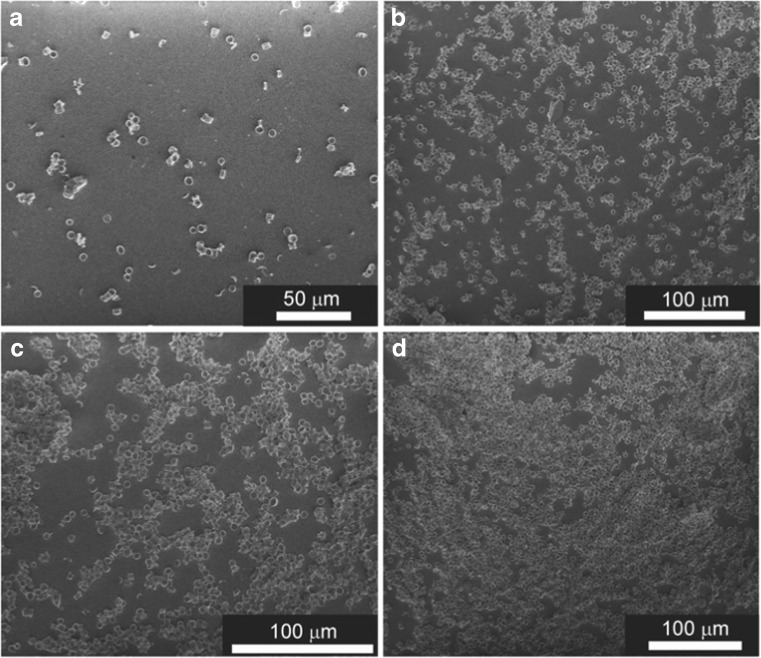
**a–d** SEM images of glass slides covered with **a** 100 μL, **b** 300 μL, **c** 800 μL, and **d** 1000 μL of a 0.5 mg mL^−1^ diatom frustule solution

In order to optimize the performance of the SERS-based immunoassay for sensing IL-8, immune platforms with different densities of diatom frustules on the surfaces of the glass slides were trialled. Figures 4a–d are SEM images of slides with 100, 300, 800, and 1000 μL of the diatom frustule solution (0.5 mg diatoms per mL of ethanol), respectively, which show how the glass slides differ in frustule coverage.

It is clearly apparent that very dense frustule packing was obtained by drop-casting multiple coats of the 800 μL of 0.5 mg mL^−1^ frustule solution onto the glass substrate.

Subsequently, the SERS activities of the DTNB-labeled immune Au nanoparticles were examined. The successful binding of the DTNB and IL-8 antibodies to the Au nanoparticles was demonstrated by UV experiments (ESM Fig. S4). As shown in Fig. S4a in the ESM, the as-received gold nanoparticle solution has a strong extinction maximum at 609 nm. This wavelength is indicative that the individual nanoparticles average approximately 70 nm in diameter. The spectrum of AuNPs redshifted a little after coating them with the Raman reporter (ESM Fig. S4b), as the LSPR band of AuNPs is very sensitive to the refractive index of the surrounding medium. The redshift of the surface plasmon resonance peak [38] demonstrates the successful binding of DTNB to the gold nanoparticles. After modification with IL-8 antibodies, there was a large decrease in the strength of this band and it redshifted from 609 nm to 637 nm, which indicates that residual surface vacancies of DTNB-labeled AuNPs are occupied by anti-IL-8 antibodies (ESM Fig. S4c). Similar results have been observed and detected using the ATR/FTIR technique [39]. Additionally, according to the literature [40], the broadening and redshift may also indicate aggregation.

**Fig. 5:**
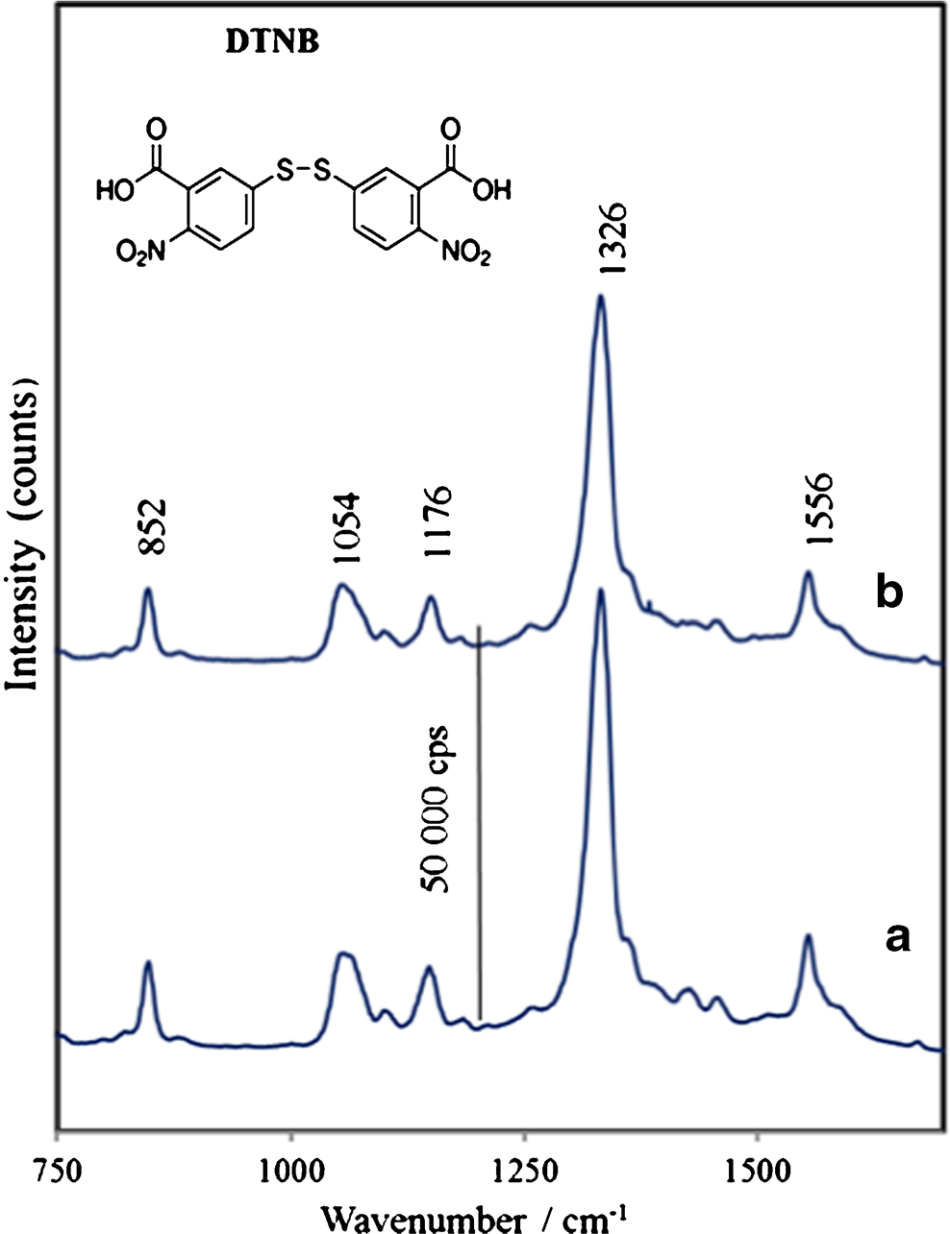
**a** SERS spectrum of DTNB (Raman reporter) adsorbed onto Au-NPs, and **b** SERS spectrum of DTNB-labeled immune-AuNPs

Figure 4a shows the SERS spectrum of DTNB chemisorbed onto AuNPs, which is very intense and dominated by bands at 1326 cm^−1^ and 1556 cm^−1^ arising from a symmetric stretching mode of nitro groups *ν*_s_(NO_2_) and an aromatic ring stretching mode, respectively [41]. The peaks at 1176 and 1054 cm^−1^ were attributed to CH_3_ rocking, C–N stretching, and C–N bending [42]. Similarly, a strong characteristic SERS signal from DTNB (Fig. 5b) was obtained for the DTNB-labeled immune Au nanoparticles (after the anti-IL-8 antibody immobilization), indicating that DTNB acts as a sensitive Raman reporter for SERS immune sensing.

### SERS immunoassay detection of IL-8

**Fig. 6:**
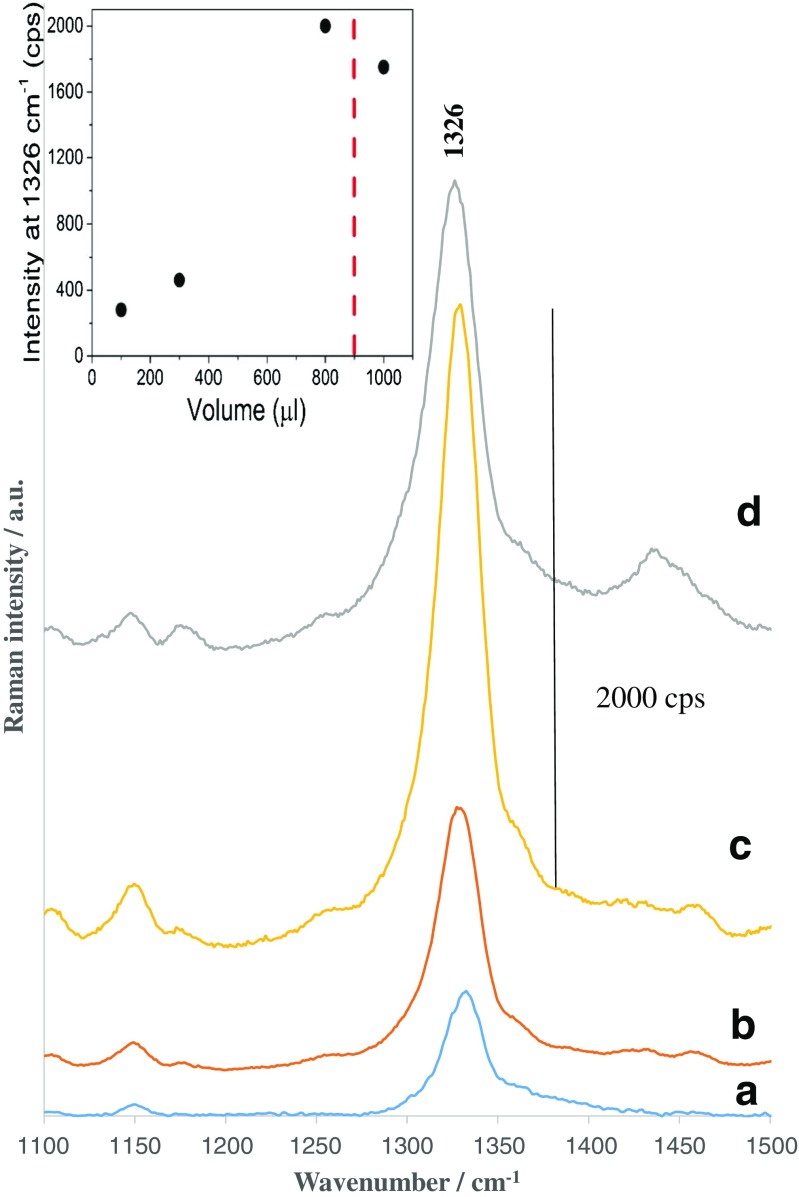
**a–d **SERS spectra of the marker band at 1326 cm^−1^ with different densities of diatom frustules on the surface of the glass slide: **a** 100 μL, **b** 300 μL, **c** 800 μL, and **d** 1000 μL of a 0.5 mg diatoms per mL of ethanol solution. The *inset* presents the relationship between the intensity of the marker band at 1326 cm^−1^ and the density of diatom frustules on the surface of the glass slide

In a first step, the four substrates with different frustule coverages (see SEM images in Fig. 4) were evaluated for their immune sensitivities according to the immunoassay protocol outlined above. All capturing surfaces were coated with anti-IL-8 antibodies and then exposed to a preselected concentration of antigen (25 ng mL^−1^ in blood plasma). The SEM images in Fig. S6 (see the ESM) demonstrate the presence of DTNB-labeled immune Au nanoparticles on the diatom frustules on the substrates. Figure 6 shows how the intensity of the most prominent band (the marker band) of DTNB at 1326 cm^−1^ changes with the coverage of the capturing substrate by the diatom biosilica. As the biosilica density increases on the surface of the glass, the SERS signal of the marker band becomes stronger (Fig. 6a–c). The red line in the inset of Fig. 6 shows the critical density of diatoms on the surface. The SERS signal in Fig. 6d does not show any further increase due to the formation of the multilayer of diatoms and aggregates, which is not as effective at immunosensing (see ESM Fig. S5 for a SEM picture of the SERS platform obtained using 1 mL of the solution of diatoms). We found that this multilayer structure of diatom-based immune substrate exhibited a significant decrease in physical/chemical stability. To examine the strength of the immobilization of the diatoms on the glass, a Scotch tape test was performed. The morphologies of the two types of capturing substrates corresponding to monolayer and multilayer arrangements of diatom frustules (see Fig. 4c and d) were observed using SEM before and after placing Scotch tape on the substrates and then peeling them off. The multilayered immune substrate was removed almost completely from the surface of glass, while the monolayer of diatom frustules was only slightly affected by the tape. These experiments indicated that the morphology and the strength of bonding of the diatom frustules with the glass substrate are important influences on the stability and sensitivity of the immunosensor.

These experimental results prove that the morphology of the capturing substrate presented in Fig. 4c is best suited to achieving the highest IL-8 detection sensitivity, and so this substrate was applied to a quantitative analysis of IL-8 in plasma blood samples.

### Quantitative analysis

**Fig. 7:**
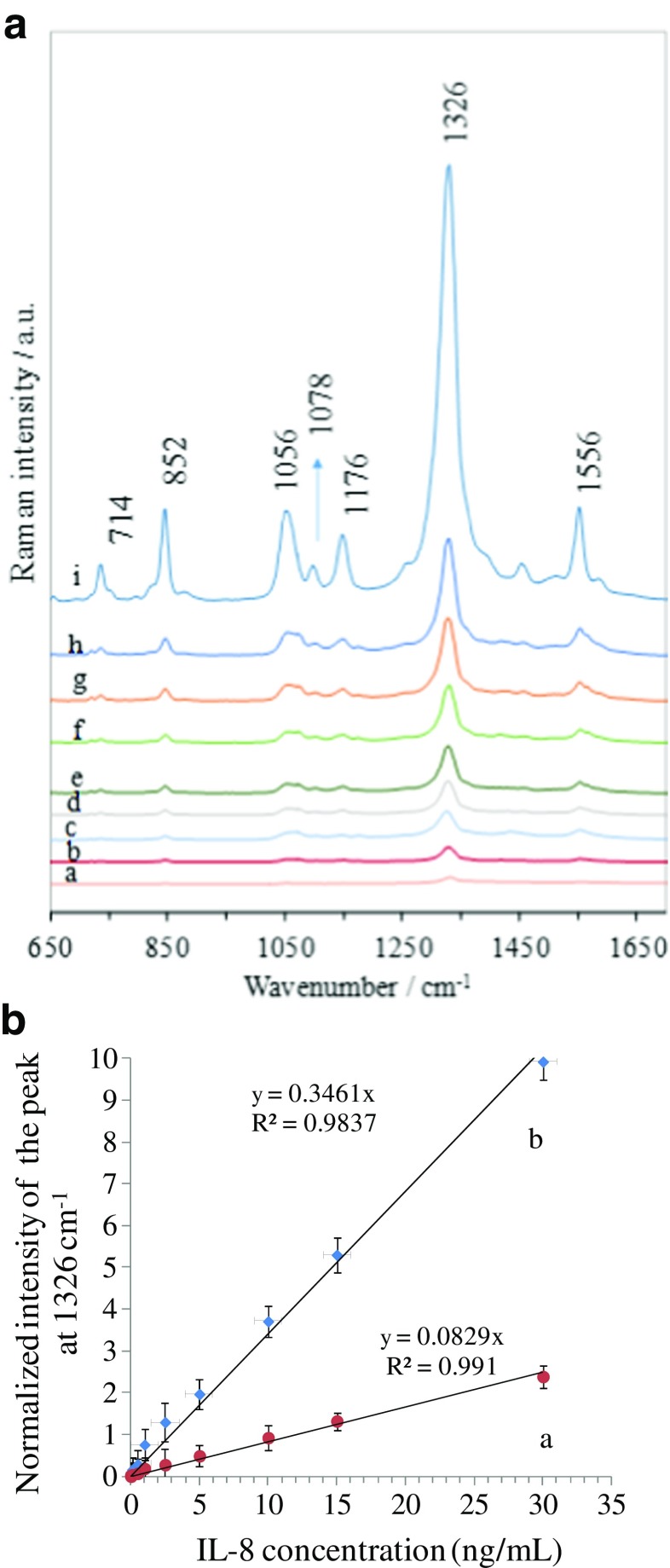
(**A**) SERS spectra obtained for various concentrations of IL-8: (**a**) 0.0; (**b**) 0.005; (**c**) 0.01; (**d**) 0.1; (**e**) 0.5; (**f**) 1.0; (**g**) 2.5; (**h**) 5.0; (**i**) 30.0 ng mL^–1^ in blood plasma. (**B**) Relationship between the normalized intensity of the marker band at 1326 cm^−1^ and the concentration of IL-8 in the range from 0 to 30 ng mL^–1^ for (**a**) a glass slide with no diatom frustules and (**b**) a glass slide with diatom frustules deposited on its surface. Each SERS spectrum is the average of 20 measurements obtained at different points on the SERS substrate surface using the mapping mode. Each *error bar* indicates the standard deviation calculated from 20 measurements obtained at different spots on the surface for a particular IL-8 concentration

The applicability of the developed SERS-based immunoassay to the quantitative analysis of IL-8 was evaluated by measuring the SERS response for varying concentrations of the target IL-8 in blood plasma samples. A dilution series of IL-8 in human blood plasma covering the range 0–30 ng mL^−1^ was prepared. This concentration range was chosen to cover clinically relevant IL-8 concentrations. Figure 7a presents SERS spectra obtained for selected concentrations of IL-8 after completing the immunoassay protocol described earlier in this paper. The intensity of the average SERS fingerprint spectrum of the Raman reporter (DTNB) increased linearly with the concentration of the target IL-8 in the sample over the whole concentration range tested. Note that, in order to achieve reproducible data, the confocal Raman mapping mode was employed to solve any problems with a nonuniform spatial distribution of SERS nanoparticles (aggregations) on the SERS substrate. A blank spectrum (Fig. 7a) was recorded by analyzing a blood plasma sample from a healthy subject without any added IL-8 antigen. When the complementary antigen is not added, the Raman-reporter-labeled immune Au nanoparticles should be easily removed from the substrate by washing. The presence of a weak peak at 1326 cm^−1^ highlights the high sensitivity of this assay, as IL-8 is present in normal healthy blood at picoogram concentrations [32]. Calibration curve (a) in Fig. 7b was obtained by plotting the normalized intensity of the SERS signal of the DTNB marker band at 1326 cm^−1^ against the concentration of the antigen in the range from 0 to 30 ng mL^−1^. Each error bar indicates the standard deviation determined from ten measurements at different spots for a particular concentration. In the linear region, the best-fit equation for the calibration curve was *y* = 0.3462*x* and the coefficient of determination (*R* ^2^) was 0.98. For the linear calibration curve, it was assumed that the SERS intensity at 1326 cm^−1^ (*y*) is linearly related to the concentration of the target interleukin (*x*). In addition, the low detection limit (LOD) was estimated using the signal-to-noise (S/N) method [43]. A S/N ratio of three is generally employed to estimate the LOD. Based on these data, the low detection limit was calculated to be 6.2 pg mL^−1^.

In a first step, a control experiment was performed to verify the specificity of the immunological recognition. The unspecific Akt blocking peptide for anti-IL-8 antibody was employed in the same immunoassay protocol as described for Fig. 2. The Akt blocking peptide specifically binds only to the Akt (pan) Rabbit mAb antibody. As can be seen in Fig. S8a of the ESM, after the Akt blocking peptide was added, there were no strong bands, only two extremely weak bands at about 840 cm^−1^ and 1326 cm^−1^ originating from the Raman reporter molecules. This may indicate that a small number of DTNB-labeled immune AuNPs were adsorbed onto the capturing substrate without any immune recognition. However, more importantly, the presence of these marker bands demonstrates that this technique can detect the low levels of IL-8 that are usually present in normal control blood plasma samples. When the anti-IL8/AuNPs-DTNB were incubated with the IL-8 in the plasma samples, strong signals originating from the Raman reporter molecules appeared in the SERS spectrum (ESM Fig. S7b–d). As can be seen in Fig. S7 of the ESM, the difference in intensity between the SERS signals from DTNB that were obtained in the specific and unspecific recognition regimes clearly demonstrates the high specificity of this immunocomplex.

In our study, we also compared the efficiency of the developed SERS immunoassay with that of a conventional SERS sensor based on flat glass. The glass slide was employed in the same immunoassay protocol as described earlier in this paper. Based on the constructed SERS response curve (b) shown in Fig. 7b, the lowest detectable concentration was 2.5 ng mL^−1^, which is two orders of magnitude worse than that obtained for the diatom-frustule-based SERS immune substrate. Although the reason for this enhanced sensitivity of the technique developed here requires further study as it is a rather complex phenomenon, it is very likely due to the unique properties of the diatom frustules.

Diatoms are photonic crystals that can increase the local field intensity, leading to Raman signal enhancement [44] and thus improving the detection limit of the SERS-based immunoassay sensor. The periodic two-dimensional pore arrays permit guided-mode resonances (GMRs) at visible wavelengths. Theoretical and experimental investigations indicate that the electric field amplitude of localized surface plasmons (LSPs) for nonmetallic structures can be significantly amplified through the coupling of the LSPs with the plasmonic structure of the diatom [45]. As a result, the presence of the diatom frustules lowers the detection limit for IL-8 to 6.2 pg mL^−1^, which is two orders of magnitude better than that achieved with a conventional glass-based SERS- immunoassay. Apart from the sensitivity, there are also other advantages of the SERS-based immunoassay sensor based on diatoms. The porous morphology of the diatom frustules leads to unique physical and chemical properties of the frustules, such as rapid mass transport inside the microchannels and outstandingly high surface area [46]. In the SERS-based assay reported here, this large surface area can magnify the number of hotspots and adsorption sites for the analyte compared with planar glass or metallic nanostructures [47], thus improving the immunoassay sensing. The abundant hydroxyl groups on their surfaces make diatom frustules very hydrophilic in comparison with a flat glass slide. Their highly hydrophilic surfaces with highly ordered nanopores of diatoms can drive the liquid flow from the glass towards the diatom frustule as a result of capillary forces [48]. In effect, the highly hydrophilic and porous diatom frustules provide a driving force that concentrates the target molecules, which may lead to a reduction in the detection limit of several orders of magnitude [49].

The current detection limit for IL-8 using a conventional ELISA test is about 15.6 pg mL^−1^ [50], which does not always meet the requirements of clinical diagnosis. Our method improves on that detection limit and potentially permits the quantitative detection of interleukins in complex biological fluids.

**Fig. 8:**
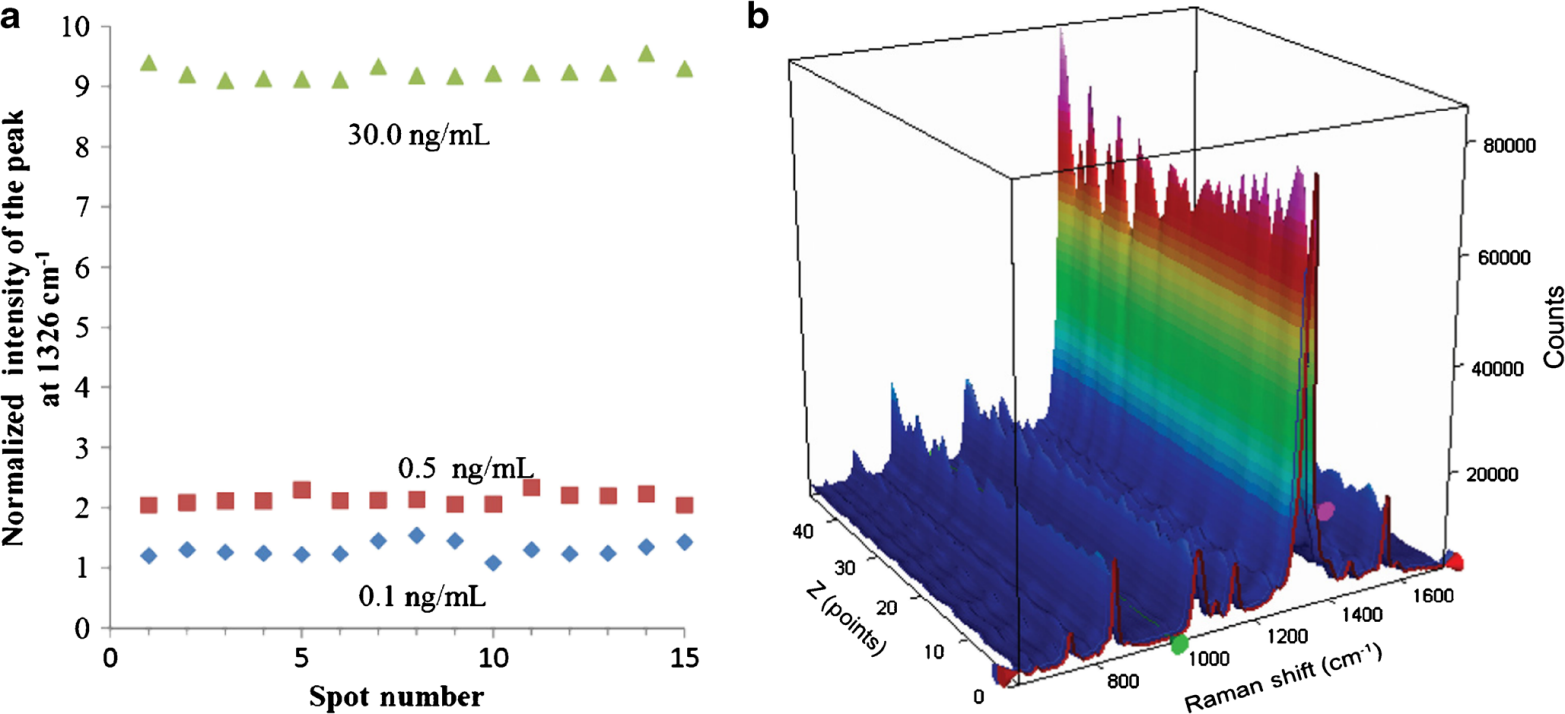
**a** Reproducibility of three separately prepared SERS immunoassays exposed to different concentrations of IL-8 in blood plasma (0.1, 0.5, and 30.0 ng mL^−1^). SERS spectra were recorded at 15 randomly selected spots on the substrate in each SERS assay. **b** Representative two-dimensional SERS spectra recorded in SERS assays of 30.0 ng mL^−1^ IL-8 performed at 40 different spots on the SERS surface. The spectra were collected over a distance of 1 mm in 10 μm steps (40 spectra are shown). Each point on the map was recorded using 5 mW of excitation at 632 nm and an integration time of 10 s

The reproducibility of the presented SERS immunoassay for IL-8 detection was also investigated. Figure 8 shows 15 individual readings from 15 randomly selected spots on three different biosilica-based SERS surfaces that were immersed in human blood plasma samples with different concentrations of IL-8 (0.1, 0.5, and 30.0 ng mL^−1^). To get a statistically valid result, the marker band of the Raman reporter at 1326 cm^−1^ was utilized to calculate the relative standard deviation (RSD). The corresponding relative standard deviations were 9.5, 8.0, and 7.2%, respectively. The relative average standard deviation (RSD) of this method was found to be less than 9%, which is comparable to that of a conventional ELISA assay.

## Conclusions

We have demonstrated, for the first time, a SERS-based immunoassay that utilizes naturally created photonic biosilica to detect the interleukin IL-8 in human plasma samples.

The most notable features of the developed SERS immunoassay include (i) the creation of an immune substrate based on anti-IL-8-functionalized diatom biosilica and (ii) the application of appropriately designed Raman-reporter-labeled AuNPs, which produce very strong SERS enhancement. The DTNB-labeled immune AuNPs can form a sandwich structure with the antigen and the antibody, as shown by the characteristic spectrum of the Raman reporter molecules (DTNB). The estimated lower detection limit and average standard deviation of the selected marker band for IL-8 at 1326 cm^−1^ show that the method is highly sensitive to clinically relevant concentrations of this interleukin and has excellent reproducibility, which is desirable for analytical analysis. This SERS assay also exhibits high biological specificity for the detection of IL-8 in complex fluids. Furthermore, the experimental results confirm that diatom frustules amplify the sensitivity of the immunosensor in comparison to a conventional sensor based on flat glass. The detection limits for IL-8 on diatom frustules and when using the glass-based SERS immune substrate in human blood plasma were found to be 6.2 pg mL^−1^ and 2.5 ng mL^−1^.

The SERS immunoassay presented here can be used for the sensitive and selective detection of immune markers in biological fluids and for point-of-care analysis.

## Electronic supplementary material

ESM 1 (PDF 2233 kb)
