# Supplementary material for: Ultrasensitive SERS immunoassay based on diatom biosilica for detection of interleukins in blood plasma
Source: Anal Bioanal Chem. 2017 Aug 29;409(27):6337–47. doi: 10.1007/s00216-017-0566-5 (PMC5641273; doi:10.1007/s00216-017-0566-5)
Supplement: Supplementary file 1 — (PDF 2233 kb) [file 216_2017_566_MOESM1_ESM.pdf]

Analytical and Bioanalytical Chemistry

Electronic Supplementary Material

Ultrasensitive SERS immunoassay based on diatom biosilica for detection of interleukins in blood plasma

Agnieszka Kamińska, M. Sprynskyy, Katarzyna Winkler, Tomasz Szymborski

1. **Synthesis of Au nanoparticles**

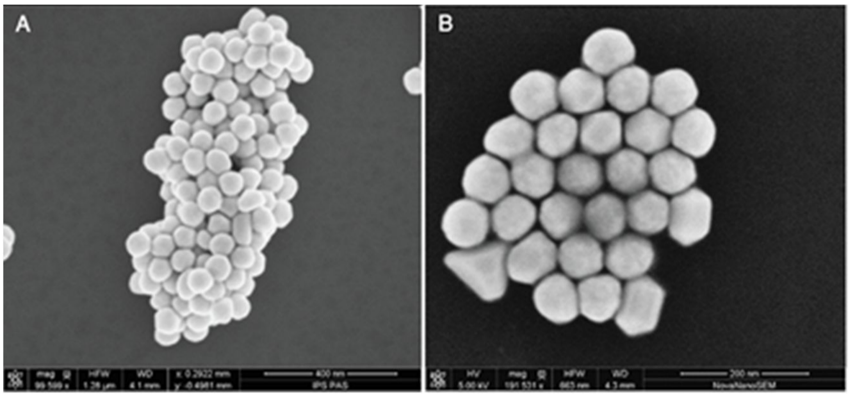

Fig. S1 The SEM images of Au nanoparticles at different magnifications (AuNPs@CTAB)

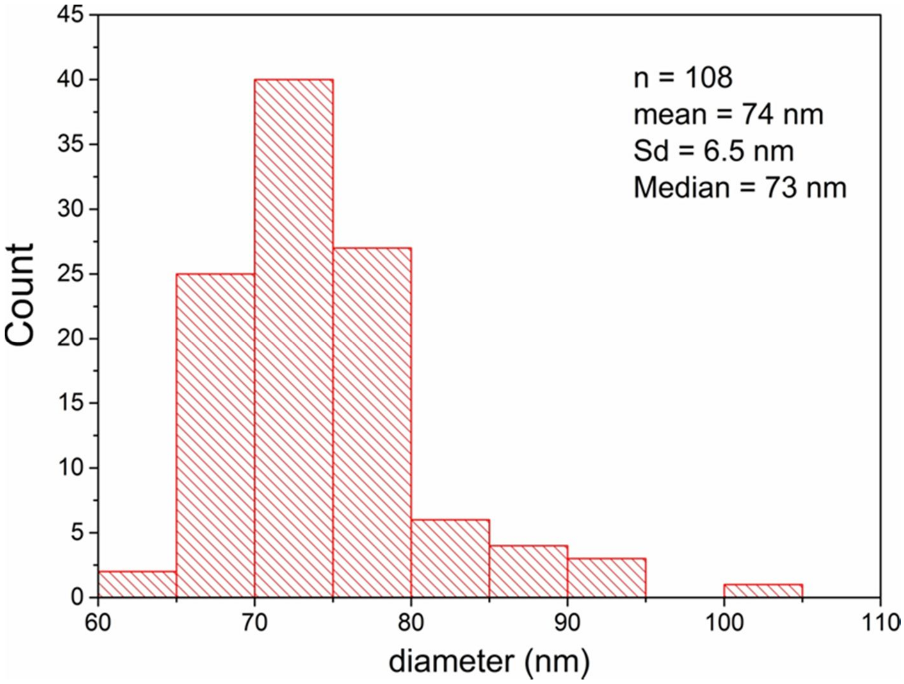

Fig. S2 Histogram of diameters of Au nanoparticles

2. **Capturing substrate and Raman reporter-labeled immune-Au nanoparticles characterization**

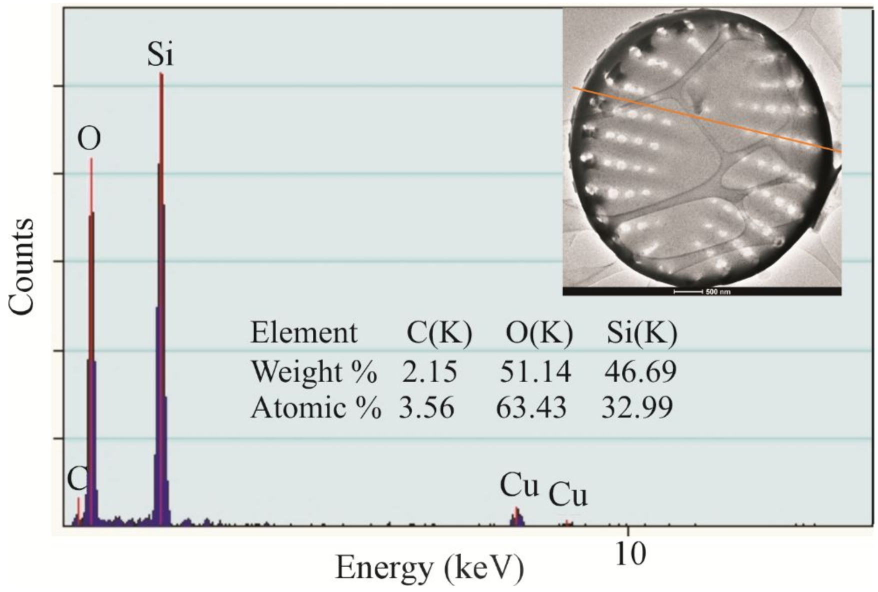

Fig. S3 TEM-EDX spectrum of elemental composition of the diatom biosilica.
The average diameter of gold nanoparticles was about 70 nm according to the SEM images (Figs. S1 and S2)

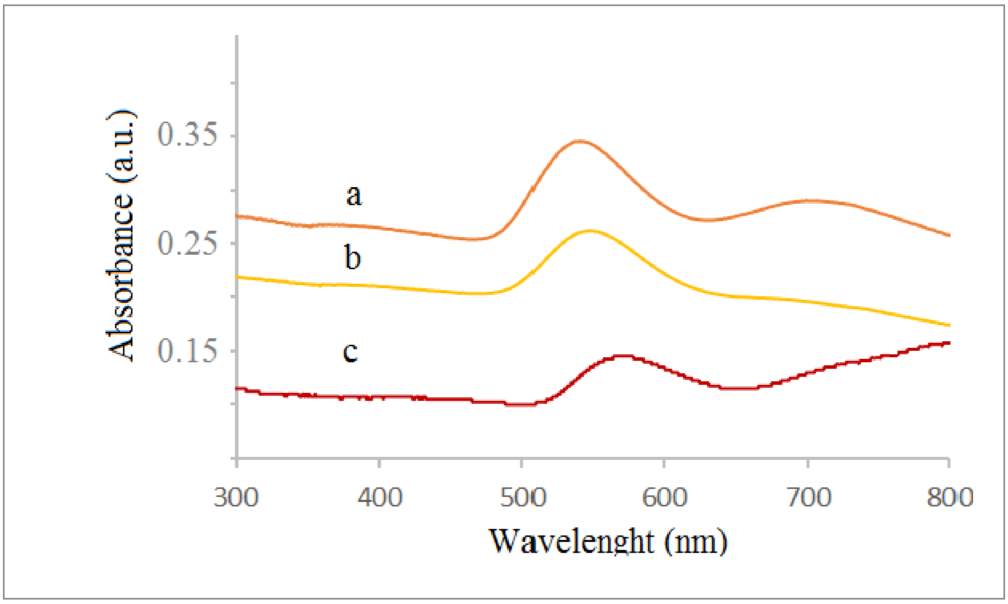

Fig. S4 The UV-visible extinction spectra of AuNPs at different steps: (a) AuNPs as-received, (b) after modification with Raman reporter (DTNB) and (c) after mixing with antibody

3. **Capturing substrate and Raman reporter-labeled immune-Au nanoparticles characterization**

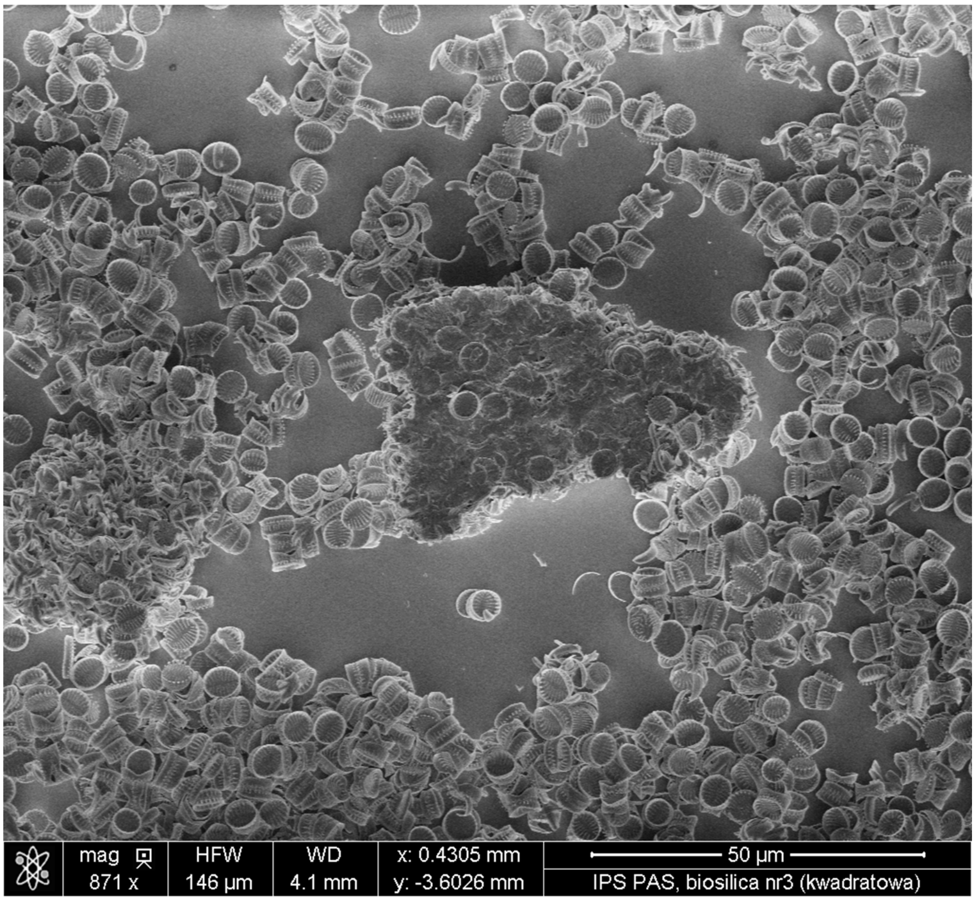

Fig. S5 SEM picture of SERS platform obtained from 1 ml solution of diatoms

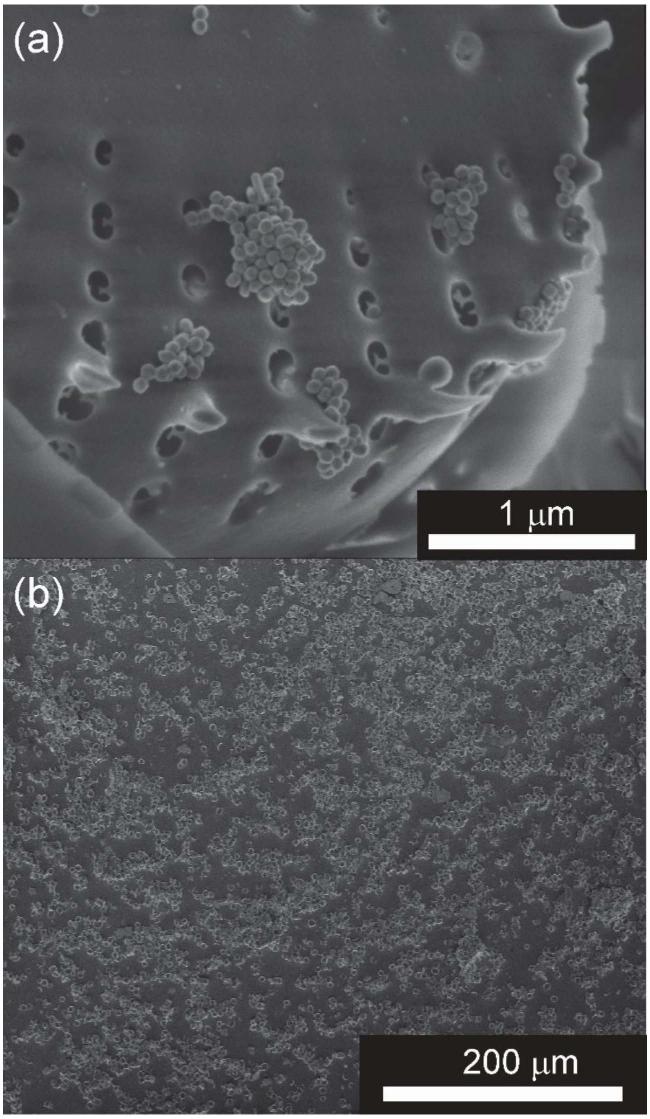

Fig. S6 (a), (b) SEM images of diatoms-based SERS immunoassay with Raman reporter-labeled immune-Au nanoparticles at different magnifications

4. SERS immuno-sensing

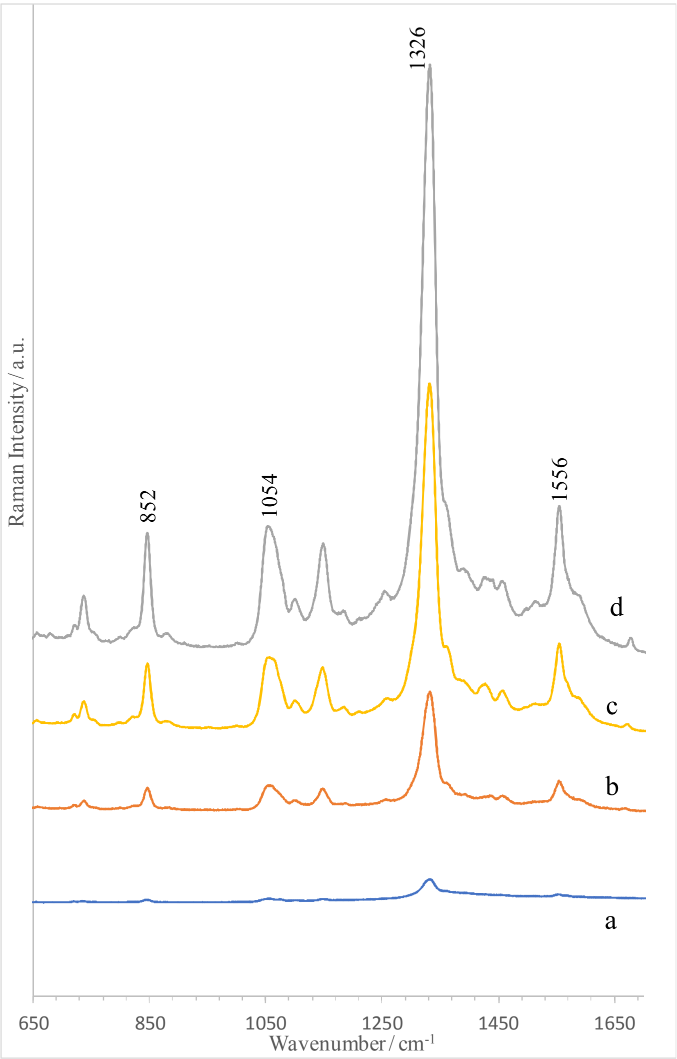

Fig. S7 (a) SERS spectrum obtained in the control experiment for 20 ng/mL Akt blocking peptide solution in blood plasma; (b, c, and d) SERS spectra in the presence of IL-8, at 5, 10, and 20 ng/mL in blood plasma solutions
